# Cultural Adaptation and User Satisfaction of an Internet-Delivered Cognitive Behavioral Program for Depression and Anxiety Among College Students in Two Latin American Countries: Focus Group Study With Potential Users and a Cross-Sectional Questionnaire Study With Actual Users

**DOI:** 10.2196/63298

**Published:** 2024-11-15

**Authors:** Yesica Albor, Noé González, Corina Benjet, Alicia Salamanca-Sanabria, Cristiny Hernández-de la Rosa, Viridiana Eslava-Torres, María Carolina García-Alfaro, Andrés Melchor-Audirac, Laura Itzel Montoya-Montero, Karla Suárez

**Affiliations:** 1 Center for Research in Global Mental Health Instituto Nacional de Psiquiatría Ramón de la Fuente Muñiz Mexico City Mexico; 2 Singapore Institute for Clinical Sciences (SICS), Agency for Science, Technology and Research (A*STAR) Singapore Singapore; 3 Universidad Nacional Autónoma de México Mexico City Mexico

**Keywords:** culturally competent care, mental health, digital health, student health services, Colombia, Mexico, SilverCloud, anxiety, depression

## Abstract

**Background:**

To scale up mental health care in low-resource settings, digital interventions must consider cultural fit. Despite the findings that culturally adapted digital interventions have greater effectiveness, there is a lack of empirical evidence of interventions that have been culturally adapted or their adaptation documented.

**Objective:**

This study aimed to document the cultural adaptation of the SilverCloud Health Space from Depression and Anxiety program for university students in Colombia and Mexico and evaluate user satisfaction with the adapted program.

**Methods:**

A mixed methods process was based on Cultural Sensitivity and Ecological Validity frameworks. In phase 1, the research team added culturally relevant content (eg, expressions, personal stories, photos) for the target population to the intervention. In phase 2, potential users (9 university students) first evaluated the vignettes and photos used throughout the program. We calculated median and modal responses. They then participated in focus groups to evaluate and assess the cultural appropriateness of the materials. Their comments were coded into the 8 dimensions of the Ecological Validity Framework. Phase 3 consisted of choosing the vignettes most highly rated by the potential users and making modifications to the materials based on the student feedback. In the final phase, 765 actual users then engaged with the culturally adapted program and rated their satisfaction with the program. We calculated the percentage of users who agreed or strongly agreed that the modules were interesting, relevant, useful, and helped them attain their goals.

**Results:**

The potential users perceived the original vignettes as moderately genuine, or true, which were given median scores between 2.5 and 3 (out of a possible 4) and somewhat identified with the situations presented in the vignettes given median scores between 1.5 and 3. The majority of comments or suggestions for modification concerned language (126/218, 57.5%), followed by concepts (50/218, 22.8%). Much less concerned methods (22/218, 10%), persons (9/218, 4.1%), context (5/218, 2.3%), or content (2/218, 0.9%). There were no comments about metaphors or goals. Intervention materials were modified based on these results. Of the actual users who engaged with the adapted version of the program, 87.7%-96.2% of them agreed or strongly agreed that the modules were interesting, relevant, useful, and helped them to attain their goals.

**Conclusions:**

We conclude that the adapted version is satisfactory for this population based on the focus group discussions and the satisfaction scores. Conducting and documenting such cultural adaptations and involving the users in the cultural adaptation process will likely improve the effectiveness of digital mental health interventions in low- and middle-income countries and culturally diverse contexts.

## Introduction

Anxiety and depression treatment gaps are particularly pronounced in low- and middle-income countries (LMICs) [1]. Many digital mental health interventions have been developed and evaluated in high-income countries and show promising results for reducing the treatment gap due to their scalability, lower human resource costs, and reduction of barriers to access [2,3]. However, LMICs and Latin America, in particular, lag behind high-income countries in developing and testing digital mental health interventions [4-6]. University students in LMICs have high rates of these disorders and digital literacy and internet access through their universities, making them ideal for such interventions [7,8].

Jordans and Kohrt [9] prioritize relevance to population needs and cultural and contextual fit in their roadmap for scaling up mental health care in low-resource settings. Most digital mental health interventions, while based on universal cognitive behavior strategies, are developed in high-income countries, which are not representative of the rest of the world [10]. So, examples and presentation of materials may need cultural sensitivity for best results. One meta-analysis of cultural adaptations of psychological interventions found 4.68 greater odds of psychopathology remission with culturally adapted interventions over other conditions [11].

Culture influences values, customs, and therapeutic practices [12], so systematic changes to intervention protocols are important for clinical trials [13]. Cultural adaptation frameworks assess intervention component selection, adaptation, piloting, and implementation [14]. Cultural adaptation in psychotherapy includes how content is transmitted, such as translation dialects or culture-specific illustrations, without regard to therapeutic components [15]. According to Resnicow et al [16], these are surface structure or top-down adaptations. The Ecological Validity Framework (EVF) [17,18] was one of the first to be used for Latin America. It involves dimensions like language, persons, metaphors, content (values and custom), concepts (constructs used), goals, methods, and context to be taken into account in the intervention to increase validity. According to the Cultural Sensitivity Framework (CSF) [19], surface and deep structure are the main dimensions for cultural adaptation. Surface structure involves matching the interventions’ materials and messages to the target population’s likenesses, names, brands, language, etc, while deep structure involves how cultural, contextual, and historic factors affect how the target population views mental disorders’ cause and treatment.

An intervention’s cultural sensitivity may affect effectiveness and user satisfaction. User satisfaction can be defined as user opinions of the system including the perceived advantages and disadvantages [20]. Implementing web-based psychological programs in the target population requires user satisfaction evaluation. Feedback from users has improved internet-delivered treatments [21,22]. While user satisfaction is likely due to many diverse factors (eg, ease of use and attractiveness) and not solely to cultural sensitivity, user satisfaction increases with culturally adapted internet-based interventions. For example, Hadjistavropoulos et al [23] found that adapting personal stories (vignettes) in an internet-delivered cognitive behavioral treatment for anxiety and depression made users feel less alone and showed how the program’s skills could be applied to real-life problems. User satisfaction and reduced posttreatment anxiety, but not depression, were correlated with this specifically targeted content.

Previously, the SilverCloud Health’s Space from Depression program [24] was culturally adapted [25] and tested [26] for Colombian university students. The adaptation used the surface approach of Resnicow et al [19], EVF of Bernal et al [17], and Helms’ [27] cross-cultural principles. As a result of the cultural sensitivity adaptation, the name was changed to “Yo Puedo Sentirme Bien,” meaning I can feel good, which was considered more culturally sensitive to the stigma attached to depression in Colombian culture. Language, personal story vignettes, and audio and video recordings were modified. In a randomized controlled trial (RCT), the culturally adapted program was found to have a large posttreatment effect size that was maintained at three months compared to a waitlist control group [26].

Since then, SilverCloud Health (now Amway Health) updated and expanded Space from Depression into a transdiagnostic Anxiety and Depression program aimed at the general adult population [28]. This program can be self-guided or guided with asynchronous weekly written feedback from a supporter, but all intervention content is identical. This internet-delivered intervention provides cognitive behavioral strategies (behavioral activation and cognitive restructuring) through 7 core modules with explanations and exercises (also called tools, such as a thoughts-emotions-behavior cycle or activity programming to be filled out by users) which are exemplified with the personal stories of 6 characters. We culturally adapted this transdiagnostic version (including elements from the previous depression-specific version for Colombian students) for both Colombian and Mexican university students and used it in a recent RCT [29,30]. This article describes the culturally sensitive adaptation of this new transdiagnostic internet-delivered cognitive behavior therapy (i-CBT) for anxiety and depression for Colombian and Mexican university students and evaluates user satisfaction.

## Methods

### Overview

We used qualitative (for COREQ [consolidated criteria for reporting qualitative studies], refer to Table S1 in Multimedia Appendix 1) and quantitative mixed methods approaches to analyze cultural sensitivity using a top-down approach based on EVF and CSF and evaluate user satisfaction with the adapted version of the SilverCloud Health Space from Depression and Anxiety program among university students in Colombia and Mexico. A total of four phases were implemented: (1) preliminary adaptation by the research team, (2) assessment of the preliminary adaptation by potential users through quantitative ratings and focus group discussions; (3) incorporation of culturally adapted suggestions, and (4) assessment of user satisfaction of the culturally adapted intervention.

### Participants

In phase 1, the research team of male and female Mexican clinical psychologists who work with university students initially adapted surface structure from the noncollege student specific transdiagnostic version. In phase 2, a convenience sample of 9 students from public universities in Colombia and Mexico was recruited by email, evaluated vignettes, and images, and participated in 2 web-based focus group discussions to assess cultural sensitivity (surface structure) and EVF. Participants were aged 19-41 years old (mean age 26.2 years). The first focus group included 6 Mexican university students (potential users), 3 male and 3 female. The second focus group included 3 Colombian university women, 1 of which had also studied in Mexico and thus, had unique experience in both contexts. In phase 3, the same research team incorporated the cultural suggestions provided in the previous phase. In phase 4, 765 college students (mean age 21.4, SD 2.9 years; 621, 81.2% female; 466, 60.9% originating from Mexico and 299, 39.1% from Colombia) were recruited as part of a clinical trial to reduce anxiety and depression [29,30]. They were recruited by one of three methods: (1) students seeking help at their university mental health clinic and on the waitlist for services were offered participation in the study; (2) email invitations were sent to randomly selected students from the list of matriculated students provided by each university; and (3) social media dissemination of the study at each university allowed students to request participation. Students meeting inclusion criteria (18+ years of age; scores of 10+ on the Patient Health Questionnaire-9 or Generalized Anxiety Disorder-7 Scale) and without exclusion criteria (current suicidal risk as determined by suicidal ideation and some intention; screen positive for bipolar disorder; report of nonaffective psychosis) were randomly assigned to one of 2 digital intervention arms (guided or self-guided) rated how interesting, relevant, useful, and helpful for obtaining their goals they found the final adapted intervention.

### Measures

Before each focus group meeting, participants completed a Google Forms questionnaire with 6 questions, 4 on a 4-point Likert scale (from “not at all” to “completely”), to assess familiarity and identification with the program’s personal stories (vignettes) and photos. The questions examined cultural sensitivity in language expressions, persons, and context. “Did this story seem true or real to you?” “Has this happened to any classmate or student you know?” “How much do you identify with this situation?” “Does this person look like a classmate or student you know?” and 2 open questions: “What part of the story is unclear or could be improved?” “If you could add something about your experience or that of someone you know, what would it be? How would you describe it?”

For the focus groups, a semistructured interview guide was developed, based on the dimensions of the EVF and CSF, to probe identification with the problem and symptoms associated with mental health problems described in the vignettes and tools (like physical and mental health issues, lifestyle choices, or migration) and how much they related to or identified with specific words, phrases, and images (surface structure). Examples of some of the questions include: “What is the main problem you identify in the story?” “Do you identify any expression in the wording that is very different from how you express yourself normally?” “Are there any changes you consider necessary to make it more understandable or relatable?” “What characteristics should the photos have that would make them more identifiable as college students?”

In quantitative questionnaires, phase 4 participants were asked to rate the extent to which they agreed or disagreed (strongly disagree, disagree, agree, and strongly agree) with 4 statements at the end of each module to assess whether the module was interesting, relevant, useful, and helped obtain their goals.

### Procedure

#### Phase 1: Preliminary Adaptation of Surface Structure

The research team thoroughly evaluated the content and materials of the Spanish language general adult population version of the program, including all text, photos, vignettes, and names of characters in the vignettes. Based on their personal experience working with Latin American university students, the team was instructed to review the materials for comprehension and aspects of Mexico and Colombia that differ from high-income Anglo-Saxon contexts. To choose new names for the characters in the vignettes, Google was searched for common names in Mexico and Colombia in the years 1990 to 2000. From a photo bank, images were chosen to represent Latin American students with neutral facial expressions that were then purchased by the SilverCloud platform.

#### Phase 2: Assessing the Preliminary Adaptation

Because of the vast amount of program material, in this phase we focused on the evaluation of the vignettes (and the photos that accompany the vignettes) as they are used throughout the program to enhance personal connection and provide examples for all of the exercises. University students from Colombia and México, were emailed a link to a Google Forms questionnaire to assess their perception of the vignettes and images as real and relatable. These same students then participated in one of 2 focus groups on Zoom led by a male and a female Mexican clinical psychologist with whom they had no previous relationship to explore the deep structure and identify anxiety and depression-related expressions, emotions, and thoughts. Members of the focus group were shown a power point presentation that combined the previously adapted depression-specific vignettes for Colombian university students with transdiagnostic vignettes that were not specific to university students, as well as instances of the platform tools using the same personal stories. For each vignette or tool, the semistructured questions were posed, and each focus group member was given the opportunity to comment. Focus group discussions were recorded and transcribed verbatim.

#### Phase 3: Modifying the Preliminary Adaptation

The research team reviewed the quantitative ratings and qualitative participant statements from the focus group discussions and selected the highest-rated vignettes, linguistic adaptations, photos, and names for the final vignettes and made modifications based on participant suggestions.

#### Phase 4: Assessing the Final Adapted Intervention

College students from Colombia and Mexico were recruited between March 2021 and May 2023 as part of an RCT to reduce symptoms of anxiety and depression [29,30]. Participants were randomly assigned to the guided and self-guided versions of the program, used the program according to their needs either through the web platform or through the program app, and assessed at the end of each module they used how interesting, relevant, useful, and helpful for obtaining their goals they considered the module to be.

### Data Analysis

To assess familiarity and identification and given the small sample size of the prefocus group questionnaires, we calculated the median and mode ratings of each vignette. Focus group verbatim transcripts were divided into participant statements for analysis. A deductive content analysis was performed, specifically focusing on the 8 dimensions of the EVF (language, persons, metaphors, content, concepts, goals, methods, and context). Statements where 7/9 of the research team disagreed on the dimension code were reviewed again by the whole team, and a consensus was reached. Each suggested modification and its rationale was considered by the research team before deciding to modify.

To evaluate user satisfaction with the adapted modules overall and with each module, we calculated the percentage of students that responded that they agreed or strongly agreed that the module was interesting, relevant, useful, and helped them obtain their goals.

### Ethical Considerations

All procedures followed relevant laws and institutional guidelines and were approved by the Research Ethics Committee of the Instituto Nacional de Psiquiatría (in Mexico) (CEI/C/015/2020). All participants provided web-based informed consent, and mental health referrals were made as necessary.

## Results

### Phase 1: Preliminary Adaptation of Surface Structure

As a result of the comprehensive review of the intervention, the original character names (Allison, Hanna, Jonathan, Juliana, Sean, and Victor) were replaced by Valentina, Carolina, Alejandro, Daniela, Santiago, and Sebastian. In addition, the research team changed all character images in the vignettes to look more Latin and like university students.

Since many of the original vignettes were about working adults with children, some storylines were changed to make them more relevant for university students. For example, Sean’s personal story said: “My wife and I bought our first house and moved in just before our daughter Isabel was born. Everything was going great. Then I injured my back in a work accident and my world turned upside down,” which was replaced by Santiago’s personal story that says, “I hoped life would be better when I got to college. I had a very bad time in high school. I come from a small town and I remember that from a very young age I felt ‘different.’ When I became a teenager, I realized that I was not physically attractive because of being overweight and other physical characteristics. For years in high school, I was bullied and made fun of. Thus, my self-esteem was completely broken. I felt like a weirdo and didn’t know how to fit in.” Similar adaptations were made for program exercises that referenced their stories.

Finally, other contextual modifications were made; for example, the “Help” section was modified with emergency telephone numbers specific to Colombia and Mexico. Also, videos explaining the platform and technological aspects were added and users were given the opportunity to request a Zoom (Zoom Video Communications) video session if they had questions.

### Phase 2: Assessing the Preliminary Adaptation

Table 1 shows the scores the students gave each original vignette before the focus group. The students generally perceived the vignettes as real and relatable given median and mode overall scores between 2.5 and 3 (out of a possible 4) and somewhat identified with the situations presented in the vignettes with scores between 1 and 3. The lowest-scoring vignettes were about an older student returning to college and a student with a serious physical illness whereas the highest-scoring ones were about relationship issues and perfectionism.

Table 2 displays examples of the feedback given by the students in the focus groups by the EVF category assigned to the statement. The majority of comments or suggestions for modification concerned language 57.5% (126/218; ie, substituting 1 word for a more commonly used synonym); followed by concepts 22.8% (50/218). Much less were regarding methods 10% (22/218); persons 4.1% (9/218); context 2.3% (5/218); and content 0.9% (2/218) and none were metaphors or goals. Many of the comments regarding language concerned words that are more or less common in Mexico or Colombia, words that are more formal or less likely to be spoken by students, whereas many of the comments regarding concepts concerned how emotions are expressed.

**Table 1:** Focus group participants’ ratings of the original vignettes.

Vignette	Did it seem true or real?			Has it happened to any classmate you know?			How much do you identify with the situation?			Does this person look like a classmate or student you know?			Overall	
	Median^a^	Mode^a^	Median^a^		Mode^a^	Median^a^		Mode^a^	Median^a^		Mode^a^	Median^a^		Mode^a^
										
Perfectionism	3	3	3		3	2		2	2		2	2.5		3
Self-esteem	3	3	3		3	2		2	2		2	3		3
Acculturation	3	3	3		3	2		2	2		2	3		3
Illness	2.5	3	1		1	1		1	1		1	1.5		1
Anxiety	3	3	3		3	2		3	2		2	3		3
Independence	3	3	2		3	1.5		1	2		2	2		3
Older student	3	3	1		1	1		1	1		1	1		1
Isolation	3	3	2		3	1.5		1	2		2	2		2
Maternity	3	3	3		3	2		3	3		3	3		3
Relationship problems	3	3	3		3	3		3	3		3	3		3

^a^The scale ranged from 1 “not at all” to 4 “completely.”

**Table 2:** Example focus group comments by Ecological Validity Framework dimension.

Dimension	Definition		Examples	Data (n=218), n (%)
Language		The suitability of the language used (whether it is “formal,” “informal,” or “jargon”) based on the context and comprehension of participants	*As a student, I wouldn't understand the technical term “cognitive behavioral therapy” if I wasn’t studying psychology or a psychologist, I wouldn't know what it means. The word “cognitive behavioral therapy” disturbs me, so I wouldn’t be able to tell a friend, “Hey, why don’t you start cognitive behavioral therapy?” As a student, I would say “psychological therapy,” I would also say “therapy” on its own.* *In Colombia, saying “comer” and “cenar” means the same thing, but “cenar” isn't used in everyday speech unless you're saying “voy a comer” or “almuerzo” in the middle of the day. It's like when you don't know a neutral text from another country, but if they want to make it local, they'd say “No como”and I go to sleep.*	126 (57.5)
Persons		Commonalities and ethnic or racial distinctions presented in the vignette	*I also remember that girl with curly hair and that guy who I think wore a red plaid shirt. He looked fine in that outfit—it was a lot like how we dress.* *That heavy person wasn’t even obvious to me as Latino. He looked like he was from another country, like England. As an example, there was a girl who was black and a guy who was white. This doesn’t happen very often in Mexico, but it does happen a little more now and then. As she comes to the city from another town, the girl looks more Latin. Although she is not as light-skinned, she is a little more familiar or looks like my classmates.*	9 (4.1)
Content		Cultural values, customs, or traditions depicted in the vignette	*I believe that the “choral ensemble committee” should be changed to something a little more up to date. I mean, people do join “feminist groups,” “paintinggroups,” and “reading groups,” but “choral ensemble” doesn't sound very contemporary to me.* *If necessary, I would say “the death of a parent” or “a friend” or something similar, but I think you need to indicate “someone very close.”*	2 (0.9)
Concepts		The problem presented in the vignette is appropriately conceptualized and understood in alignment with the culture, context, belief system, or expectations of the participants	*I understand that the person in the vignette feels “overwhelmed” or more like “depressed” like she “feels a lot of pressure on top of her”. It´s not exactly that you get “depressed” so much as feeling “confused” or “afraid”. Something like that, no?* *For me, the moment he says that someone hurt him, you can connect with the facts or the situation, since bullying is a common problem that the story talks about. I believe it is very well organized that it is a problem from the past and that it just gathers ideas from earlier times. It is something that keeps it together; I believe you can find it in any situation.*	50 (22.8)
Methods		The efficacy and suitability of the vignette is in accordance with the participants or culture	*I personally feel like I can relate to the vignette. Because I'm older than the people in my university, I don't belong to their generation. It makes me feel like I don't fit in and that I can't make friends like you would with someone your age, but because of generational differences, I feel like I'm living apart or experiencing university differently because they are eighteen years old and are doing things for the first time.* *I think what keeps me from being able to explain it well is that the story doesn't make it clear what happens with his health. Okay, if he's sick, that's it. But I think the problems you have will depend on the illness he has, so I don't think it's clear what's wrong with him.*	22 (10)
Context		Social, economic, or political factors such as gender, acculturation, migration, or poverty that are identified in the vignette	*I believe the issue lies with the context, the activities, and being in a totally new life situation.* *Not only is the older person’s behavior a problem, but so is ours as first- year college students. We don't know how to interact with older people because we often see them as superior, so we don't joke around or get along with them. I think this happens a lot. Regarding the story, I believe I am confused because I believe you can go back to school when you lose your job, but it is also true that you can’t do that right now. If the economy is really bad, I don't think you would really go back to school.*	5 (2.3)
Miscellaneous		Typographical or editing errors	*It was hard for me to follow this story because it said “daughter” and then “son” at different times, making me wonder if it was daughter or son.* *It talked about Haiti, the Dominican Republic, and the United States, so I couldn't figure out where it was.*	4 (1.8)

### Phase 3: Modifying the Preliminary Adaptation

To retain only the number of needed vignettes and as a result of the prefocus group questionnaire and focus group discussions, the vignettes about an older student returning to college and a student with a serious physical illness were eliminated. First, because they scored lowest on the questionnaire about the perception of authenticity. Second, because in the focus group discussions, students mentioned ecological validity issues in EVF methods.

For example, in the vignette about an older student returning to college, a participant said:

And specifically, about the story, I think I have a confusion because I think that you can go back to studying when you lose your job, but it is also true that the current context does not allow you to do so. If the economic stability is really very bad, I don't know if you would really come back to study.

In the case of the vignette of the student with a serious physical illness, the students reported that they could not identify with the story because it was too ambiguous and because of the formal wording. For example, a student said:

I think that what prevents me from being able to relate to it well is the fact that what is happening with his health is left very vague. Ok, if he is sick, that's it, but I think that depending on the illness he has, he will have different difficulties, so I feel like it leaves what happens to him very vague.

While another said:

Well, with this one it happens more to me than with the others, I also think that the way it is worded seems very unrealistic to me, you would never say I entered university or in spite of, I think I would make it a little less formal.

In the rest of the vignettes, the main changes occurred in the language and concept categories of EVF. In the language category, changes were made to less formal or more common expressions for university students from Colombia and Mexico. For example, a participant commented that “The phrase “the fatigue was too much” sounds very formal; “I'm tired” would be more appropriate.” And so, the text was changed to: “I was very tired.”

In the concepts category, changes were made according to cultural values, customs or traditions for university students from Colombia and Mexico, many regarding how emotions are expressed. For example, in the original version, the word “hurt” was used, but this concept was changed to “upset,” because a participant expressed:

We don't usually speak like that. I would say “I'm upset or distressed” instead of “I'm hurt,” as I relate it to a physical injury.

Another example of changes that were made due to concepts was: “I moved to the city because there were no universities in the community where I was born.” This was replaced by: “I moved to the city because there were no universities in the region where I was born.” “Region” was used instead of “community” because a Colombian participant reported that upon reading “community”:

I assumed it was a reference to an individual from an indigenous group. If you’re looking for a more general term to refer to someone who moved out of a municipality, you could use “área” or “región.

For Mexico, both concepts, region or community, are understood.

Students also made comments like “The ideas are exactly in line with what I would say or what I have heard from peers.” For vignettes or descriptions with comments such as these no changes were made.

### Phase 4: Assessing the Final Adapted Intervention

In Table 3, we show the user ratings of the intervention modules (which used the modified vignettes) by module and by program modality. Overall, 91.2% (698/765) to 95.6% (731/765) agreed or strongly agreed that at least one module was interesting, relevant, useful, and helped them attain their goals with ratings ranging from 87.7% (607/692) to 96.2% (127/132) for individual modules. The number of students who explored each of the modules is also shown since the program is not linear, nor is it necessary to review all the modules and students were able to benefit from just one module. As can be seen in the table, the introductory module (getting started) was the most reviewed by far.

**Table 3:** User ratings of intervention modules.

Module		Participants who rate each of the modules, n	Interesting, n (%)^a^	Relevant, n (%)^a^	Useful, n (%)^a^	Helped to obtain goals, n (%)^a^
Overall		765	731 (95.6)	704 (94.1)	698 (91.2)	709 (92.7)
**Core modules**						
	Getting started	692	652 (94.2)	630 (91)	607 (87.7)	615 (88.9)
	Understanding feelings	379	354 (93.4)	353 (93.1)	340 (89.7)	342 (90.2)
	Boosting behavior	277	259 (93.5)	255 (92.1)	254 (91.7)	256 (92.4)
	Spotting thoughts	204	187 (91.7)	185 (90.7)	188 (92.2)	187 (91.7)
	Challenging thoughts	165	152 (92.1)	154 (93.3)	151 (91.5)	151 (91.5)
	Managing worry	132	125 (94.7)	127 (96.2)	124 (93.9)	124 (93.9)
	Bringing it all together	82	76 (92.7)	75 (91.5)	74 (90.2)	75 (91.5)

^a^Sum of “strongly agree” and “agree.”

## Discussion

### Principal Findings and Comparison With Previous Work

There is a dearth of reports on cultural adaptations of digital mental health interventions despite reports that culturally adapted interventions have greater treatment effects [31]. This study systematically describes a theoretically driven cultural sensitivity adaptation, considering dimensions such as language, people, context, content, etc., of the Space from Depression and Anxiety i-CBT intervention into a culturally sensitive and ecologically valid version called Yo Puedo Sentirme Bien (“I can feel good”). Roughly 90% of actual users were satisfied with the culturally adapted version.

In a first stage, the research team conducted a preliminary adaptation of the surface structure of the intervention vignettes, tools, and photos. We tailored the internet-delivered program (eg, language), while maintaining the original treatment components (fidelity). In a second stage, potential users (Colombian and Mexican university students) participated in further tailoring of vignettes, tools, and images. Their comments and suggestions were coded according to the 8 EVF dimensions. Most of the changes suggested by the students concerned language (substituting a word for another more commonly used synonym) and concepts (particularly how emotions are expressed and how the problems are understood) rather than changes in the facts or experiences of the vignettes (ie, context and content). Perhaps because in the first stage adaptation visibly culturally dissonant attributes of other dimensions were already modified. The feedback from potential users in this study was similar to that of the feedback from potential users in a cultural adaptation of the SilverCloud depression only program for Colombian students [25], with comments centered on the language used (particularly making it more colloquial), though they also commented the relevance of examples and personal stories and conceptual clarity. While the current study did not evaluate quantitatively the linguistic, functional and conceptual equivalence, the aforementioned study [25] reported user ratings of 4.2, 4.5, and 4.6 for linguistic, functional and conceptual equivalence, respectively, on a scale of 1 to 5. Another culturally sensitive adaptation of a different intervention (keepin’it REAL) for a different intervention target (substance use prevention) [32] used a similar adaptation process based on CSF and EVF and similarly coding adaptations into the 8 EVF dimensions.

Based on this second stage, the research team selected the vignettes and photos that the potential users identified with the most (through their preliminary ratings of the vignettes and photos and their spontaneous comments in the focus groups like “I personally feel like I can relate to the vignette”). Also, the team made modifications to the chosen vignettes from the suggestions of the potential users. Finally, user satisfaction with the program was rated by a much larger independent group of actual users.

We found support for satisfaction with the adapted program by actual users (Colombian and Mexican university students) participating in an RCT. Around 90% found the adapted modules interesting, relevant, useful and helpful for obtaining their goals. While we cannot determine from this study whether culturally adapting the intervention increased satisfaction, our satisfaction ratings are similar or better than those reported by university students in other studies. For example, our findings are higher than the 69% of students in a large university in the United States that found the (original English) program helpful or very helpful [33] and similar to the results of an i-CBT for college students with depression, anxiety, or insomnia, of whom 87.5% were somewhat or very satisfied with the program [34] and a study of acceptability of an internet-delivered intervention for highly stressed Dutch university students, in which satisfaction of users who completed core sessions had a mean of 25.8 (on a scale from 8-32) indicating good satisfaction [35].

### Study Limitations

A possible limitation of the study is that the research team that conducted the phase 1 adaptation were all Mexican and there were more participants from Mexico than Colombia in the focus groups. Given that the program was previously culturally adapted and tested for Colombian college students (by coauthor [ASS]), which may have attenuated this limitation to some extent, it was considered important to have a first focus group with Mexican students to ensure the cultural sensitivity and comprehension of language and the second focus group with Colombian students was to ensure that modifications suggested by the Mexican students were shared by the Colombian students. In addition, one of the Colombian students lived in Mexico and could corroborate shared neutral terms that could be understood in both countries.

Another limitation of the study is that participants were not obliged to evaluate each module and most did not evaluate all modules. Therefore, it is likely that users who were most satisfied with the program used and thus, evaluated more modules, whereas less satisfied users simply did not continue to another module. Thus, our estimations of satisfaction may be inflated. However, modules explored by more participants did not have a lower proportion of participants who were satisfied, and the lowest ratings were still fairly positive with more than 87% agreeing that the module was interesting, relevant, useful, and helped them to attain their goals.

The multiple and broad strategies for recruiting both help-seeking and non–help-seeking students with anxious or depressive symptoms reduces sampling bias. The students were more likely to be female which is consistent with females more likely to have anxiety and depression and more likely to seek help for their problems [8]. The findings are more likely to be biased by those not sufficiently engaged with the program to respond to the satisfaction questions at the end of each module than with the sampling procedures.

While the focus of this paper is on the cultural adaptation of the intervention materials and user satisfaction, a separate publication has shown the intervention to be effective [29]. Because uptake and engagement are an important limitation of digital mental health interventions in general [36], future research such look at how cultural adaptation of intervention materials impacts uptake and engagement.

### Study Contributions

A recent review of meta-analyses of cultural adaptations found a general lack of standardized frameworks for cultural adaptation that are routinely used in research and practice and called for further research into the process of adaptation [14]. This study adds to the small number of digital intervention studies that have conducted and described the cultural adaptation of the intervention, using surface structure from CSF, ecological evaluation from EVF, incorporating feedback from potential users and satisfaction ratings of actual users. Conducting and documenting such cultural adaptations and involving the users in the cultural adaptation process will likely improve the effectiveness of digital mental health interventions in LMICs and culturally diverse populations and settings, and thus, ultimately reduce the treatment gap. There is limited knowledge about the optimal cultural adaptation, and therefore the incorporation of user satisfaction evaluations supports the adapted program’s relevance for the population. Culturally sensitive mental health interventions are necessary for public health. This study contributes evidence for using this particular culturally adapted intervention in Colombian and Mexican university students as well as provides a guide for conducting culturally sensitive adaptations of public health interventions.

Digital interventions for mental health are proliferating especially in the high-income English-speaking countries with results that show comparable results to face to face interventions [37] at lower costs and greater reach. We show that interventions that have already been developed in these countries can be successfully culturally adapted in such a way that is acceptable to actual users in non–English speaking contexts in LMICs. In conjunction with being acceptable in this context, we have previously shown that they are also effective [29]. This suggests that political decision makers in LMICs could build upon digital mental health interventions already developed in other contexts at minimal investment.

### Conclusions

In conclusion, we illustrated the process of culturally adapting a digital mental health intervention based on cultural sensitivity and ecological validity frameworks to enable others to use similar methodologies and begin to build a consensus in the field on this process, as well as to document the cultural adaptation of a specific program, SilverCloud Health Space from Depression and Anxiety program for Colombian and Mexican university students, which may be of interest to researchers, clinicians, and policy makers in Latin America. Because there is a plethora of digital mental health interventions in English-speaking high-income countries and just beginning such development in Latin America, the choice to develop new interventions from scratch in these countries versus culturally adapt existing interventions from English-speaking high-income countries can be informed by these findings that roughly 90% of Latin American student users find the adapted version satisfactory.

## Appendix

Multimedia Appendix 1 Additional material.

## Abbreviations

COREQ: consolidated criteria for reporting qualitative studies
CSF: Cultural Sensitivity Framework
EVF: Ecological Validity Framework
i-CBT: internet-delivered cognitive behavioral therapy
LMIC: low- and middle-income country
RCT: randomized controlled trial
