# Supplementary material for: Cultural Adaptation and User Satisfaction of an Internet-Delivered Cognitive Behavioral Program for Depression and Anxiety Among College Students in Two Latin American Countries: Focus Group Study With Potential Users and a Cross-Sectional Questionnaire Study With Actual Users
Source: JMIR Form Res. 2024 Nov 15;8:e63298. doi: 10.2196/63298 (PMC11607549; doi:10.2196/63298)
Supplement: Multimedia Appendix 1 [file formative_v8i1e63298_app1.docx]

| Appendix Table 1. Consolidated criteria for reporting qualitative studies (COREQ)^1^ | | | |
| --- | --- | --- | --- |
| Topic | Item No. | Guide questions/description | Reported on Page # and/or response |
| *Domain 1: Research team and reflexivity* |  |  |  |
| Interviewer/facilitator | 1 | Which author/s conducted the interview or focus group? | Focus groups were conducted by YA and NG |
| Credentials | 2 | What were the researcher’s credentials? E.g. PhD, MD | PhD, p. 4 |
| Occupation | 3 | What was their occupation at the time of the study? | Clinical Psychologist, p.4 |
| Gender | 4 | Was the researcher male or female? | p. 4 |
| Experience and training | 5 | What experience or training did the researcher have? | p. 4 |
| Relationships established | 6 | Was a relationship established prior to study commencement? | p. 4 |
| Participant knowledge of the interviewer | 7 | What did the participants know about the researcher? e.g. personal goals, reasons for doing the research | Participants were aware that the purpose of the study was to know if the personal stories that are used as examples in an online program to reduce depression and anxiety are relevant to the university population in Mexico and Colombia and if they resemble people and situations with which they are familiar. This was included in the informed consent. |
| Interviewer characteristics | 8 | What characteristics were reported about the inter viewer/facilitator? e.g. Bias, assumptions, reasons and interests in the research topic | Participants were informed that the interviewers were professionals participating in the cultural adaptation of an online program from anxiety and depression. |
| *Domain 2: Study design* |  |  |  |
| Methodological orientation and theory | 9 | What methodological orientation was stated to underpin the study? e.g. grounded theory, discourse analysis, ethnography, phenomenology, content analysis | p.7 |
| Sampling | 10 | How were participants selected? e.g. purposive, convenience, consecutive, snowball | p.4-5 |
| Method of approach | 11 | How were participants approached? e.g. face-to-face, telephone, mail, email | p.5 |
| Sample size | 12 | How many participants were in the study? | p.4 |
| Non-participation | 13 | How many people refused to participate or dropped out? Reasons? | No refusals; No dropouts |
| Setting of data collection | 14 | Where was the data collected? e.g. home, clinic, workplace | p.5 online |
| Presence of non-participants | 15 | Was anyone else present besides the participants and researchers? | No one else present |
| Description of sample | 16 | What are the important characteristics of the sample? e.g. demographic data, date | p.4-5 |
| Interview guide | 17 | Were questions, prompts, guides provided by the authors? Was it pilot tested? | Brief description p.5, Not pilot tested. |
| Repeat interviews | 18 | Were repeat inter views carried out? If yes, how many? | Two separated focus groups conducted; No focus groups were repeated |
| Audio/visual recording | 19 | Did the research use audio or visual recording to collect the data? | p. 6 |
| Field notes | 20 | Were field notes made during and/or after the interview or focus group? | No |
| Duration | 21 | What was the duration of the inter views or focus group? | Focus group 1: 1 hour 39 minutes.  Focus group 2: 1 hour 50 minutes |
| Data saturation | 22 | Was data saturation discussed? | No |
| Transcripts returned | 23 | Were transcripts returned to participants for comment and/or correction? | No |
| *Domain 3: Analysis and findings* |  |  |  |
| Number of data coders | 24 | How many data coders coded the data? | p.7 |
| Description of the coding tree | 25 | Did authors provide a description of the coding tree? | No subthemes were coded |
| Derivation of themes | 26 | Were themes identified in advance or derived from the data? | p.7 |
| Software | 27 | What software, if applicable, was used to manage the data? | No specialized software, only Excel |
| Participant checking | 28 | Did participants provide feedback on the findings? | No |
| Quotations presented | 29 | Were participant quotations presented to illustrate the themes/findings? Was each quotation identified? e.g. participant number | p.7, p.8, Table 2 |
| Data and findings consistent | 30 | Was there consistency between the data presented and the findings? | p.7-9, Table 2 |
| Clarity of major themes | 31 | Were major themes clearly presented in the findings? | p.7, p.8, Table 2 |
| Clarity of minor themes | 32 | Is there a description of diverse cases or discussion of minor themes? | No subthemes identified, Table 2 |
| ^1^Developed from: Tong A, Sainsbury P, Craig J. Consolidated criteria for reporting qualitative research (COREQ): A 32-item checklist for interviews and focus groups. Int J Qual Health Care. 2007; 19: 349-57. | | | |
